# Targeted delivery of galbanic acid to colon cancer cells by PLGA nanoparticles incorporated into human mesenchymal stem cells 

**DOI:** 10.22038/AJP.2022.20022

**Published:** 2022

**Authors:** Mahboubeh Ebrahimian, Sanaz Shahgordi, Rezvan Yazdian-Robati, Leila Etemad, Maryam Hashemi, Zahra Salmasi

**Affiliations:** 1 *Nanotechnology Research Center, Pharmaceutical Technology Institute, Mashhad University of Medical Sciences, Mashhad, Iran *; 2 *Department of Immunology, School of Medicine, Golestan University of Medical Sciences, Gorgan, Iran*; 3 *Molecular and Cell Biology Research Center, Faculty of Medicine, Mazandaran University of Medical Sciences, Sari, Iran*; 4 *Pharmaceutical Research Center, Pharmaceutical Technology Institute, Mashhad University of Medical Sciences, Mashhad, Iran *; 5 *Department of Pharmaceutical Biotechnology, School of Pharmacy, Mashhad University of Medical Sciences, Mashhad, Iran *; 6 *Department of Pharmaceutical Nanotechnology, School of Pharmacy, Mashhad University of Medical Sciences, Mashhad, Iran*

**Keywords:** Nano-engineered mesenchymal stem cells, Targeted delivery, Cellular carrier, Galbanic acid, PLGA, Cancer

## Abstract

**Objective:**

The aim of this study was to investigate the efficacy of mesenchyme stem cells (MSCs) derived from human adipose tissue (hMSCs) as carriers for delivery of galbanic acid (GBA), a potential anticancer agent, loaded into poly (lactic-co-glycolic acid) (PLGA) nanoparticles (nano-engineered hMSCs) against tumor cells.

**Materials and Methods:**

GBA-loaded PLGA nanoparticles (PLGA/GBA) were prepared by single emulsion method and their physicochemical properties were evaluated. Then, PLGA/GBA nanoparticles were incorporated into hMSCs (hMSC/PLGA-GBA) and their migration ability and cytotoxicity against colon cancer cells were investigated.

**Results:**

The loading efficiency of PLGA/GBA nanoparticles with average size of 214±30.5 nm into hMSCs, was about 85 and 92% at GBA concentration of 20 and 40 μM, respectively. Nano-engineered hMSCs showed significant higher migration to cancer cells (C26) compared to normal cells (NIH/3T3). Furthermore, nano-engineered hMSCs could effectively induce cell death in C26 cells in comparison with non-engineered hMSCs.

**Conclusion:**

hMSCs could be implemented for efficient loading of PLGA/GBA nanoparticles to produce a targeted cellular carrier against cancer cells. Thus, according to minimal toxicity on normal cells, it deserves to be considered as a valuable platform for drug delivery in cancer therapy.

## Introduction

Despite outstanding advancement in medical technology, cancer remains one of the leading causes of mortality and morbidity throughout the world. Chemotherapy is one of the commonly used methods for cancer treatment, but some important limitations including drug resistance, failures in the chemotherapy during metastasis, insufficient tumor selectivity and cytotoxic effects on healthy tissues led to development of other strategies for cancer treatment (Charbgoo et al., 2020 ▶; Hashemi et al., 2020 ▶). 

Over the past decades, different herbal products with tremendous chemical diversity have been investigated for their anticancer properties (Huang et al., 2021 ▶). Galbanic acid (GBA), isolated from *Ferula* species (Apiaceae) has been documented to have various promising biological activities including anticancer, cell cycle arrest effects and anti-proliferative activities in different cancer cells (Sajjadi et al., 2019 ▶; Shahcheraghi et al., 2021 ▶). However, clinical application of GBA is widely limited by low solubility, low permeability in aqueous media and poor bioavailability. So, to overcome these obstacles and improve the pharmacological properties of GBA, different delivery systems have been introduced. Poly (lactic-co-glycolic acid) (PLGA) as the most prevalent nano-polymer drug carrier is a biodegradable and biocompatible polyester which has been approved by the FDA and extensively applied for delivery of different therapeutic agents including drugs, genes, proteins and peptides (Du et al., 2021 ▶; Lin et al., 2021 ▶).

Recently, Mesenchyme stem cells (MSCs) as an efficient cell-based therapy systems have attracted a great deal of attention for the targeted delivery of anticancer drugs into primary tumors and metastases (Heidari et al., 2020 ▶; Hour et al., 2020 ▶; Yin et al., 2020 ▶). Some clinical advantages such as easy isolation from multiple tissues, low immunogenic properties, fast *ex vivo* expansion, immunomodulatory functions, damage repair capacity, feasibility of autologous transplantation and ability to be manipulated or genetically modified, qualify MSCs as ideal vehicles for drug/gene delivery (Gao et al., 2013 ▶; Krueger et al., 2018 ▶). However, anticancer drug cytotoxicity on MSCs and rapid drug efflux remain significant challenges. Incorporating controlled-release nanoparticles (NPs) such as PLGA into MSCs is an alternative delivery approach to conquer these problems (Zhang et al., 2015 ▶). 

In this study, GBA-loaded PLGA NPs were constructed and then incorporated into MSCs derived from human adipose tissue (hMSC/PLGA-GBA NPs). Furthermore, the migration and cytotoxicity of hMSC/PLGA-GBA NPs against colon cancer cells were investigated.

## Materials and Methods

**Materials**

Galbanic acid (GBA) was purchased from Dr. Iranshahi, Department of Pharmacognosy and Biotechnology, School of Pharmacy, Mashhad University of Medical Sciences, Mashhad, Iran. Dulbecco’s modified eagle’s medium-low glucose (L-DMEM), fetal bovine serum (FBS), penicillin-streptomycin, phosphate-buffered saline (PBS) and trypsin were obtained from Gibco BioCult (Paisley, UK). Alizarin red and Oil red staining reagents, polyvinyl alcohol (PVA; average MW~31–50 kDa) and PLGA (Mw 7,000-17,000) were purchased from Sigma Aldrich Co (St Louis, MO, USA).

**Methods**

**Preparation of GBA-loaded PLGA NPs**

Galbanic acid-loaded PLGA nanoparticles (PLGA/GBA NPs) were prepared using single emulsion solvent evaporation technique (Hafezi Ghahestani et al., 2017 ▶). Briefly, PLGA (25 mg) and GBA (1.25 mg) were dissolved in 1 ml aceton:dichloromethane (1:4) and stirred for 15 min. The prepared solution was then added to PVA (5% w/v) as an aqueous phase under sonication on ice (amplitude 80%, for 10 min) using a probe sonicator (Fisons Instruments Ltd., Crawley, UK). The prepared emulsion was added dropwise to 10 ml of PVA 0.1%. The reaction process was continued while stirring overnight in order to evaporate the organic solvent. The NPs as final products were obtained by centrifugation (at 18000 rpm for 20 min), washed three times with distilled water to remove excess surfactant, and finally lyophilized (Hashemi et al., 2021 ▶).

**Characterization of the synthesized NPs**

Particle size (diameter (nm)) and ζ-potential (surface charge) and polydispersity index (PDI) of NPs were determined by laser light scattering (Zetasizer Nano ZS 3000 HS, Malvern, UK). The morphology of NPs was monitored using the atomic force microscope (AFM) (Park Scientific, Inc., Sunnyvale, CA).

**Determination of encapsulation efficiency (EE%) and loading content (LC%) in PLGA NPs**

For evaluating the encapsulation efficiency and GBA-loading content, PLGA/GBA NPs (1 mg) were dissolved in acetonitrile (1 ml) and sonicated for 5 min to completely degradate the PLGA matrix. After centrifugation, the supernatant was collected and the GBA concentration was measured at 325 nm by UV-Vis spectrophotometry (UV-160A, Shimadzu, Japan) (Ebrahimian et al., 2017 ▶; Afsharzadeh et al., 2019 ▶). The GBA encapsulation efficiency and the loading content were calculated via the following equations: 

LC (%) =Mass of GBA in NPs/ Mass of GBA - Loaded NPs × 100%. 

EE (%) = Amount of GBA in NPs/Amount of GBA used for encapsulation × 100%.

***In vitro*** **release of GBA from PLGA/GBA NPs**

*In vitro* release of GBA from PLGA/GBA NPs was investigated using centrifugation method. PLGA NPs suspension (200 µl), containing GBA (40 µM), were added to PBS (800 µl, pH 7.4) or citrate buffer (800 µl, pH 5.5) and incubated at 37°C, at a fixed speed of about 100 rpm. Supernatant was collected at 1, 2, 4, 24, 48, 72, 96, 120 hr using centrifugation at 17000 g for 20 min (Ebrahimian et al., 2016 ▶; Mosafer et al., 2017 ▶). After each step, the supernatant was collected and replaced with the same amount of fresh buffer to keep the buffer volume unchanged and provide sink condition. The GBA concentration was measured at 325 nm by UV-Vis spectrophotometry. Experiments were performed in triplicate and the release data is shown as the cumulative percentage of GBA with respect to the primary content of GBA in the NPs versus time. 

**Cell lines**

Mesenchymal stem cells were isolated from the adipose tissue of healthy human according to our previously published work and cultured in L- DMEM medium (Gibco, USA) containing 10% FBS (Gibco, USA), penicillin (100 IU/ml), streptomycin (100 μg/ml) (Azimifar et al., 2021 ▶). All the processes were approved by Mashhad University of Medical Sciences review committee (Approval number IR.MUMS.SP.1396.116). C26 (Mouse Colon Carcinoma) and NIH/3T3 cells were purchased from Pasteur Institute of Tehran, Iran and cultured in RPMI medium containing FBS (10%) and antibiotics (1%). All cells were cultured at 37°C in a humidified incubator containing 5% CO_2_/95% air. 

**Evaluation of hMSC surface markers by flow cytometry**

Expression of human MSC antigens (CD90 and CD44) and the absence of blood cell markers (CD45 and CD34) were assessed by flow cytometry, using a FACS Calibur instrument (Becton Dickinson) based on the manufacturer's instruction (L Ramos et al., 2016 ▶).

**Osteogenic and adipogenic differentiation of hMSCs**

The osteogenic and adipogenic differentiation potential of isolated MSCs was assessed via induction in differential osteogenic and adipogenic medium separately. The medium was changed every 2–3 days. After 21 days, osteogenic differentiation was investigated by staining the cells with Alizarin Red S solution (Santa Cruz, CA) to observe calcium-nodule deposits. For adipogenic differentiation, lipid droplets were stained by Oil Red O solution (Santa Cruz, CA) (Tayarani-Najaran et al., 2021 ▶).

**Loading PLGA/GBA NPs into hMSCs**

hMSCs (10^5 ^cells/ml) were incubated as a single cell suspension in serum-free DMEM medium with PLGA/GBA (20 and 40 µM of GBA) for 4 hr at 37^o^C. Then, hMSCs suspension was centrifuged at 1500 rpm for 5 min and supernatant was collected. PLGA/GBA NPs loading into hMSCs was determined through indirect method. For this purpose, the obtained supernatant was centrifuged at 14000 rpm for 20 min and sedimented pellet was lysed by adding acetonitrile (200 μl). Then, methanol (400 μl) was added and the mixture was centrifuged for 15 min at 17000 rpm. At the end, the final supernatant was analyzed for GBA content using UV-Vis spectrophotometry at 325 nm (Wang et al., 2018 ▶). 

**Release from nano-engineered hMSCs**

hMSCs (5 × 10^4^ cells) incorporated with PLGA/GBA NPs (20 and 40 µM of GBA) were suspended in 50 ml FBS-free DMEM medium containing 0.1% tween 80, and incubated at 37^o^C. At predetermined time points (1, 2, 4, 24, 48, 72, 96, 120 and 144 hr), cells were centrifuged at 1000 rpm for 5 min and supernatant (450 μl) was removed and replaced with the same amount of fresh medium. Collected supernatants were stored at 4°C and examined by UV-Vis spectrophotometry at 325 nm. The percentage of GBA release from engineered hMSCs at each time point was calculated and plotted as described in section 2.3.5 (Zhao et al., 2017 ▶). 

**Cytotoxicity of the synthetized NPs**

The cytotoxicity of GBA and PLGA/GBA NPs on hMSCs, C26 and NIH/3T3 cells was assessed using MTT assay. Cells (5×10^3^ cells/well) were seeded in 96-well plates and incubated overnight in a humidified incubator. Then, GBA and PLGA/GBA NPs were added at concentrations of 1.25-40 µM of GBA to hMSCs and 20-120 µM of GBA to C26 and NIH/3T3 cells. Untreated cells were used as control group. After 72 hr, cells were washed with PBS and treated with 20 µl of MTT (5 mg/ml in PBS) solution for 4 hr. The crystals formed were dissolved by 100 µL of dimethyl sulfoxide (DMSO). The absorbance was measured at 570 nm with a reference wavelength of 630 nm by an Infinite® 200 PRO multimode microplate reader (Tecan Group Ltd, Männedorf, Switzerland) (Salmasi et al., 2018 ▶; Hashemi et al., 2022 ▶). 

***In vitro*** **migration assay**

The tumor-tropism capacity of naive and engineered hMSCs was assessed using a 24-well Transwell plate (PET membrane, 8 μm pore size, Corning). In Transwell plate, C26 and NIH/3T3 cells (2 × 10^4^ cells/well) were seeded in the bottom chamber. After 24 hr, engineered and naive hMSCs (4 × 10^4 ^cells) were suspended in serum-free DMEM medium and added to the top chamber of the Transwell plate. Implanted cells were incubated at 37ºC to investigate migration through the membrane, overnight. After that, to assay the cell migration, cells remained on the upper side of top chamber were removed carefully and cells migrated to the lower side of top chambers were fixed with methanol and stained with Giemsa solution. Stained cells were observed and counted under an inverted microscope (five fields of view, at 10× magnification). Experiments were performed in triplicate (Salmasi et al., 2020 ▶). 

***In vitro*** **evaluation of antitumor activity of nano-engineered hMSCs**

The anti-tumor effect of nano-engineered hMSCs was monitored using co-culture assay. Cancerous C26 and normal NIH/3T3 cells (2 × 10^4^) were seeded at the bottom chambers of the Transwell plate (PET membrane, 0.4 μm pore size, Corning). After 24 hr, naive hMSCs and nano-engineered hMSCs at different concentrations of GBA (20, 40 and 80 µM) were added to the top chambers of the Transwell plate. Untreated cells were used as control group. After 72 hr incubation, the viability of C26 and NIH/3T3 cells was determined by MTT assay as described in section 2.2.10. 

**Statistical analysis**

Statistical analysis was conducted by GraphPad Prism 8 software (GraphPad software, CA, USA). Data are presented as means±SD of triplicates and comparison among the different groups was made by one-way ANOVA followed by Student-Newman-Keuls assuming equal variance in two groups. The level of statistical significance in all analyses was set at p<0.05. 

## Results

**Physicochemical properties of PLGA/GBA NP**

**Figure 1:**
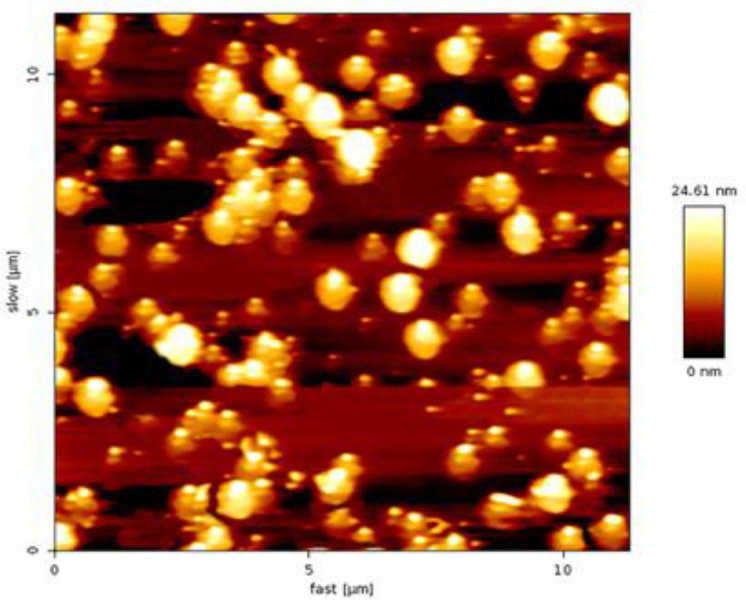
AFM image of PLGA/GBA NPs. PLGA: Poly (lactic-co-glycolic acid), GBA: Galbanic acid, NPs: Nanoparticles

Particle size, polydispersity index (PDI) and zeta potential of PLGA and PLGA/GBA are presented in Table 1. The encapsulation efficiency (EE%) and drug loading content (LC%) related to GBA into PLGA NPs were 71.2% and 3.9%, respectively. AFM image illustrated that PLGA NPs had spherical morphology with uniform distribution and average size of about 200 nm (Figure 1). 

**Table 1:** Particle size and zeta potential of PLGA and PLGA/GBA NPs (n=3 replicates for each group)

**Polydispersity index (PDI)**	**Zeta potential (mV)**	**Z-average (nm)**	**Sample**
0.277±0.035	-17.3±2.4	234.4±25.4	PLGA
0.035±0.530	-15.4± 1.2	214±30.5	PLGA/GBA

PLGA: Poly (lactic-co-glycolic acid), GBA: Galbanic acid and NPs: Nanoparticles

***In vitro*** **release of GBA from PLGA/GBA NPs**

**Figure 2:**
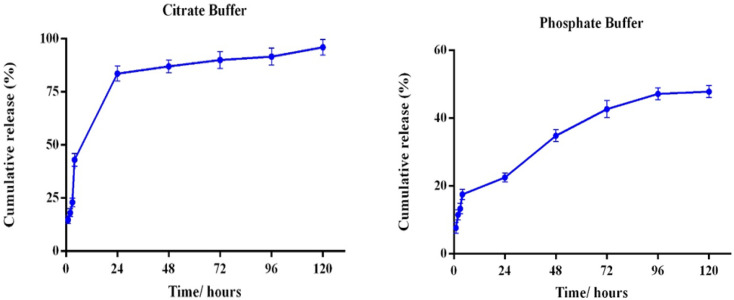
Evaluation of GBA release from PLGA/GBA NPs (40 µM, GBA-equivalent) prepared in phosphate buffered solution (PBS) and citrate buffer at different time points at 37°C. PLGA: Poly (lactic-co-glycolic acid), GBA: Galbanic acid, NPs: Nanoparticles

*In vitro* release of GBA from PLGA/GBA NPs is shown in Figure 2. GBA released from PLGA NPs (40 µM, GBA-equivalent) during the first day in PBS buffer (pH 7.4) was only 21% followed by sustained release with approximately 50% of GBA release in 120 hr. In the citrate buffer with acidic pH (which represents the acidic environment around the tumor or inside the lysosome), during the first day, 80% of the GBA loaded into the NPs was released at high speed and then the GBA release process was constant. By the fifth day, almost all of the GBA was removed from the NPs. 

**Evaluation of surface markers of hMSCs extracted from adipose tissue**

**Figure 3:**
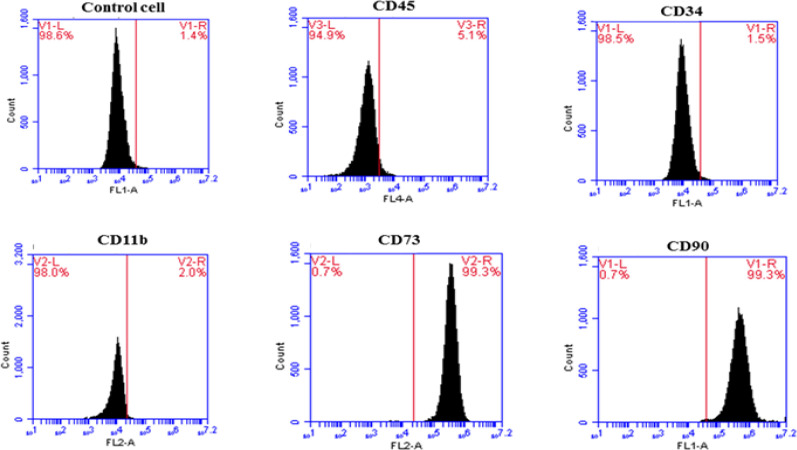
Investigation of cell surface antigens of MSCs isolated from human adipose tissue, using flow cytometry

Expression of surface antigens on extracted hMSCs (at passage 3) was studied using flow cytometry. As shown in Figure 3, these cells were positive for hMSC markers (CD44 and CD90) and negative for hematopoietic markers (CD45 and CD34). 

**Differentiation capacity of hMSCs**

**Figure 4:**
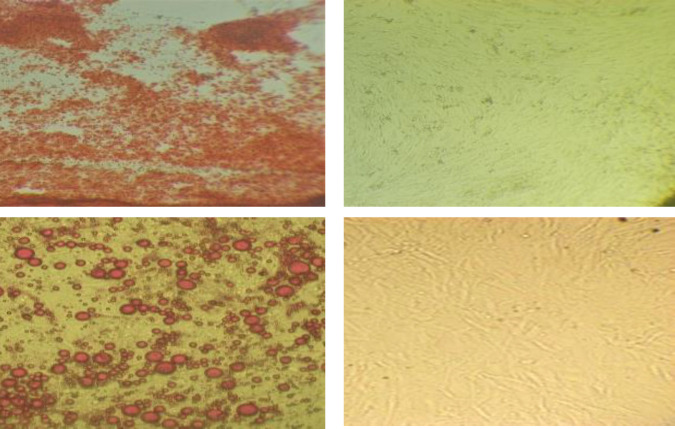
(A) Differentiation of hMSCs to osteoblasts. Calcium deposits are shown with Alizarin Red S staining in differentiated cells (left) compared to the control (right). The magnification was ×4. (B) Differentiation of hMSCs to adipocytes. Accumulation of lipid vacuoles is detected with Oil red O staining in differentiated cells (left) compared to the control (right). The magnification was ×10

Multilineage differentiation potential of isolated hMSCs at passage 3 was verified by treating them in induction mediums for three weeks. As shown in Figure 4, Alizarin Red S (Figure 4A), and Oil Red O staining (Figure 4B) revealed the successful differentiation of hMSCs.

**Loading of PLGA/GBA NPs into hMSCs**

The internalization of PLGA/GBA NPs into hMSCs suspension was explored using UV-Vis spectrophotometry at 325 nm through indirect method. The intracellular uptake was estimated about 80 and 92% for GBA 20 and 40 μM, respectively, after 4 hr incubation.

**Release from nano-engineered hMSCs**

**Figure 5:**
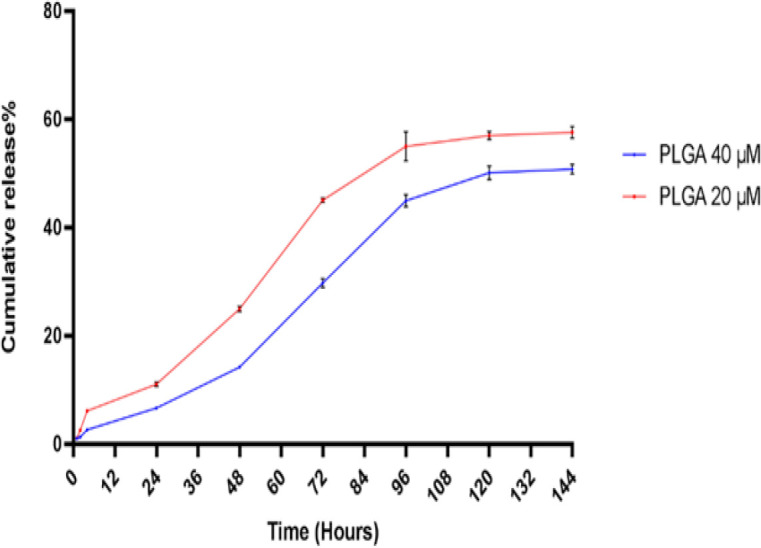
GBA released from nano-engineered hMSCs in FBS-free DMEM medium containing 0.1% tween 80 that were incubated at 37^o^C. Data are presented as mean±SD, n=3. GBA: Galbanic acid, hMSCs: human Mesenchymal stem cells, FBS: Fetal bovine serum, DMEM medium: Dulbecco’s modified eagle’s medium

As shown in Figure 5, after 72 hr, release of GBA was 45.06 and 29.80% for 20 and 40 µM GBA-equivalent, respectively followed by sustained release of GBA approximately 60% and 50% in 144 hr.

**Cytotoxicity of GBA and PLGA/GBA NPs against hMSCs, C26 and NIH/3T3 cells**

**Figure 6:**
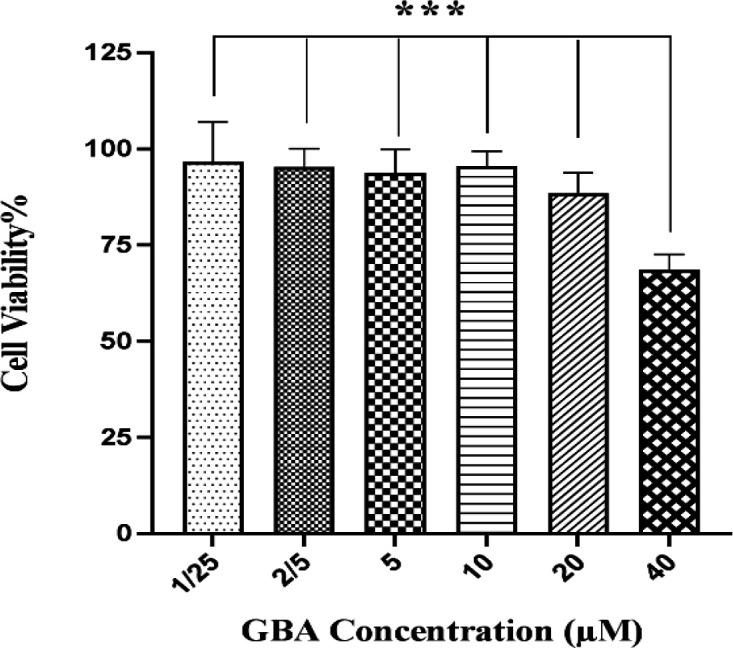
Effect of different concentrations of GBA (1.25-40 µM) on hMSCs viability after 72 hr illustrated by MTT assay. Data are presented as mean±SD, n=3, ***p<0.001. Untreated cells were used as control group. GBA: Galbanic acid, hMSCs: human Mesenchymal stem cells

**Figure 7:**
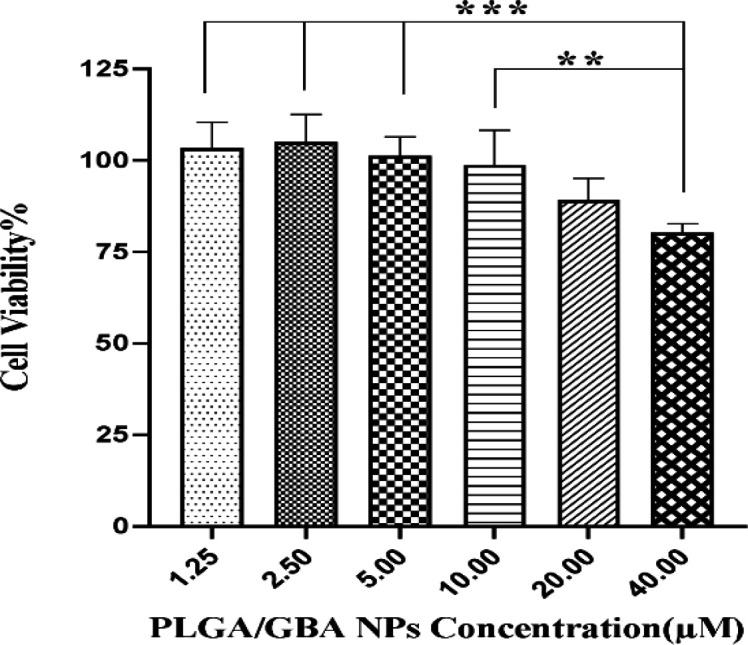
Effect of different concentrations of PLGA/GBA NPs (1.25-40 µM) on hMSCs viability after 72 hr as assessed by MTT assay. Data are presented as mean±SD, n=3. **p<0.01 and ***p<0.001. Untreated cells were used as control group. PLGA: Poly (lactic-co-glycolic acid), GBA: Galbanic acid, NPs: Nanoparticles, hMSCs: human Mesenchymal stem cells

**Figure 8:**
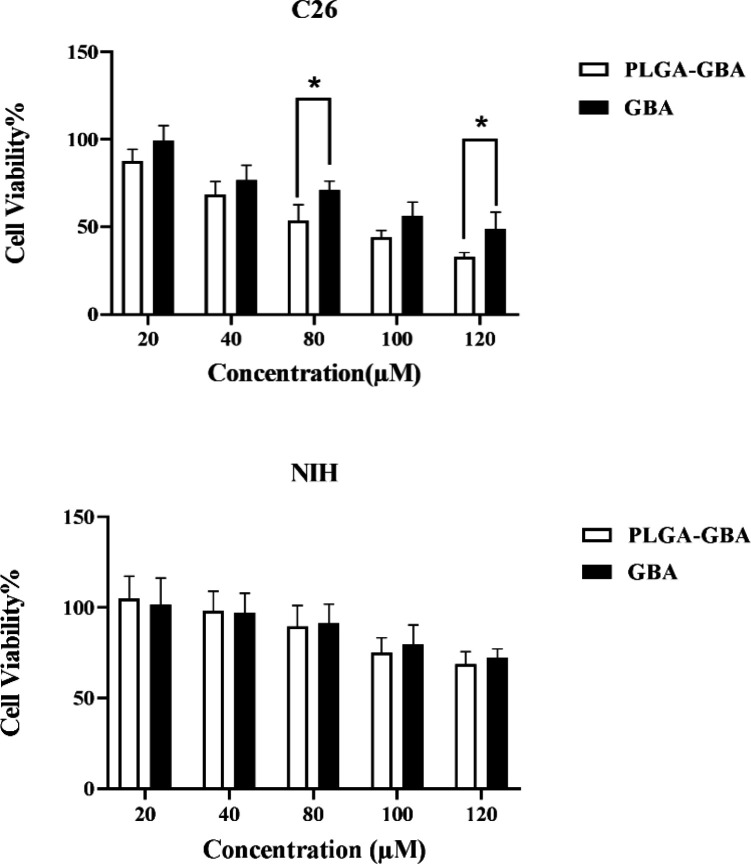
Effect of different concentrations of GBA and PLGA/GBA NPs (20-120 µM) on C26 (Mouse Colon Carcinoma) and NIH/3T3 (Mouse fibroblast) cells viability after 72 hr as assessed by MTT assay. Data are presented as mean±SD, n=3, *p<0.05. Untreated cells were used as control group. PLGA: Poly (lactic-co-glycolic acid), GBA: Galbanic acid, NPs: Nanoparticles

hMSCs viability, assessed by MTT assay, following exposure to GBA and PLGA/GBA NPs is illustrated in Figure 6 and 7 respectively. hMSCs survival after 72 hr incubation with different concentrations of GBA and PLGA/GBA (1.25-40 µM, GBA-equivalent) was not affected. At concentration of 40 µM, cell viability in GBA-treated cells decreased while hMSCs treated with PLGA/GBA showed 80% viability, suggesting that PLGA/GBA was non-toxic to hMSCs. As shown in Figure 8, significant cytotoxicity was observed for PLGA/GBA NPs, compared to GBA, at concentration of 80 and 120 µM in C26 cells. Conversely, in NIH/3T3 cells, no considerable toxicity was evaluated in any concentrations of PLGA/GBA NPs. 

***In vitro*** **tumor tropism of nano-engineered hMSCs**

To follow the migration ability of naive hMSCs and nano-engineered hMSCs towards cancerous cells, the Transwell migration assay was performed. 

**Figure 9:**
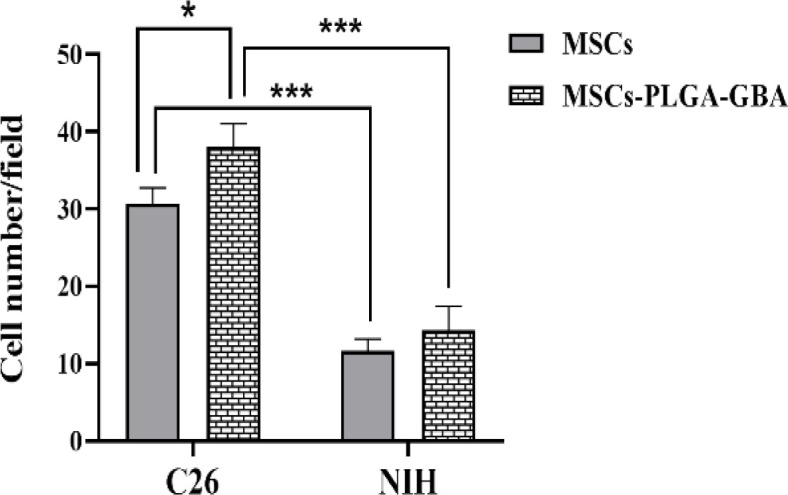
Naive and nano-engineered hMSCs migrated toward C26 (Mouse Colon Carcinoma) and NIH/3T3 (mouse fibroblast) cells. Mean migrated cells from five random fields were considered; ***p<0.001 and *p<0.05. Untreated cells were used as control group. hMSCs: human Mesenchymal stem cells

As demonstrated in Figure 9, minimal migration towards NIH/3T3 cells as normal cells was observed in treated groups. Surprisingly, migration of hMSCs loaded with PLGA/GBA NPs through the membrane pores towards C26 in the bottom chamber, significantly increased in comparison to unloaded one (p<0.05), representing the tropism of loaded hMSCs toward tumor cells. Furthermore, tropism of both naive and nano-engineered hMSCs significantly decreased toward normal cells (NIH/3T3) (p<0.001).

***In vitro*** **cytotoxicity of nano-engineered hMSCs**

**Figure 10:**
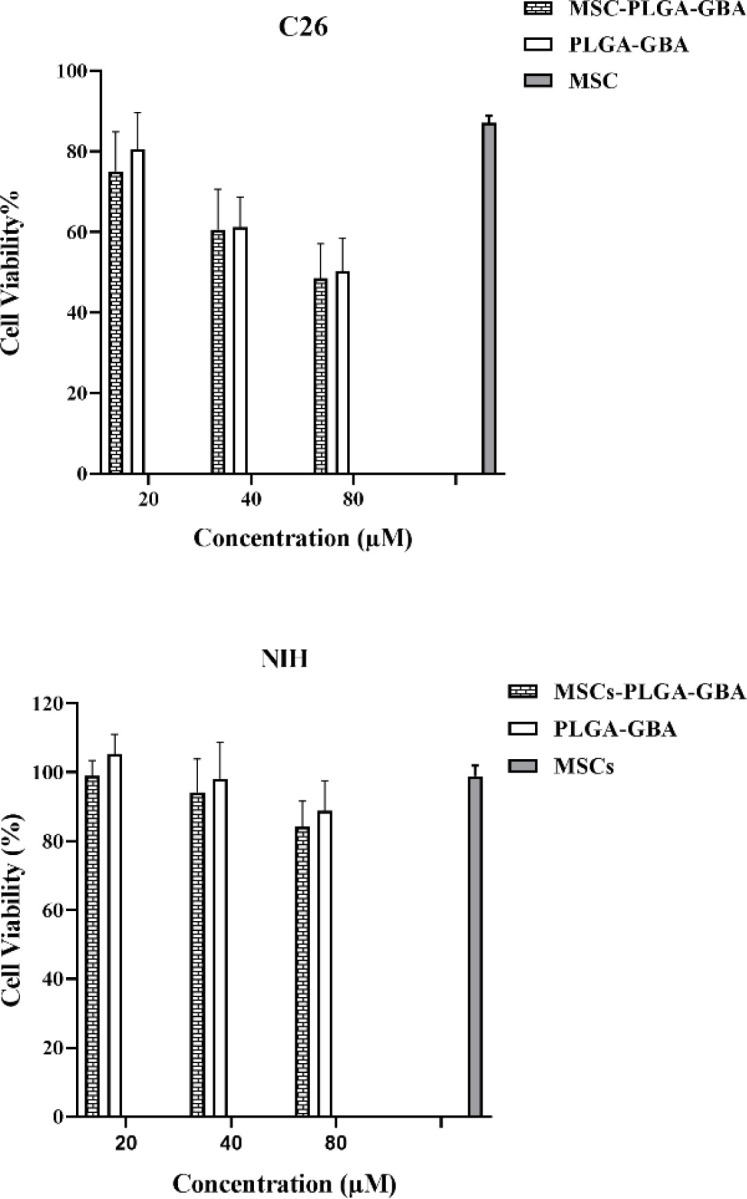
Cytotoxicity of nano-engineered hMSCs in cancer cells (C26, Mouse Colon Carcinoma) and normal cells (NIH/3T3, mouse fibroblast) following treatment with different concentrations of GBA-equivalent (20-80 µM) for 7 hr as evaluated by MTT assay. Data are presented as mean±SD, n=3. Untreated cells were used as control group. hMSCs: human Mesenchymal stem cells

To investigate the *in vitro* cytotoxic potential of nano-engineered hMSCs on tumor cells, they were added on the top chamber of a Transwell plate in concentration of 20, 40 and 80 μM GBA-equivalent, while C26 and NIH/3T3 cells were in the bottom chamber. Results of MTT analysis revealed that nano-engineered hMSCs at concentration of 40 and 80 μM could reduce the viability of C26 cells after 72 hr. Conversely, NIH/3T3 cells survival was unaffected after 72 hr incubation with different concentration of nano-engineered hMSCs. Furthermore, C26 cells viability was not affected by naive hMSCs during 72 hr implying that hMSCs as the cellular vehicle had no effect on inhibiting or promoting cancer cells growth (Figure 10). 

## Discussion

Recently, many studies have focused on the chemoprotective properties of natural products with high effectiveness and low side effects. Galbanic acid (GBA), a major lipophilic compound of *Ferula* species roots, fights progression of tumor cells via inducing G1 and G2/M arrest, inhibition of the vascular endothelial growth factor (VEGF)‐induced proliferation, and preventing hypoxia inducible factor‐1α (HIF‐1α) transcriptional activation via suppressing the EGFR/HIF‐1α signaling pathway (Kim et al., 2011 ▶; Zhang et al., 2012 ▶; Eskandani et al., 2015 ▶; Oh et al., 2015 ▶; Gharedaghi Kloucheh et al., 2021 ▶). However, poor solubility and poor bioavailability of GBA in aqueous media limited its clinical applications. Therefore, many studies have focused on development of nano-formulations for improving its therapeutic efficiency (Nik et al., 2019 ▶; Afsharzadeh et al., 2020 ▶).

Among the various approaches, polymeric carriers have been noted for their great properties including high stability and transport of both hydrophobic and hydrophilic drugs and active ingredients (Afsharzadeh et al., 2020 ▶).

Here, we used PLGA NPs to incorporate GBA, as an anti-tumor agent, in hMSCs. Biodegradable/biocompatible PLGA NPs were used with the aim of improving the solubility and chemical stability and to enhance the bioavailability of GBA (Ding and Zhu, 2018 ▶). PLGA NPs present several advantages such as being biodegradable, biocompatible, non-immunogenic and non-toxic (Semete et al., 2010 ▶). Therefore, these properties make PLGA NPs suitable for stem cell engineering.

On the other hand, low targeting efficiency of NPs restricts their applications in cancer therapy (Zhang et al., 2016 ▶). Cell-based targeting approaches using MSCs have shown potent tumor-homing potential in response to pro-inflammatory cytokines in tumor microenvironment (Chulpanova et al., 2018 ▶). Moreover, it has been documented that MSCs have low immunogenicity and a positive safety for *in vivo* studies and clinical trials (Huang et al., 2020 ▶).

As it was reported, MSCs have been engineered for delivery of chemotherapeutic drugs such as paclitaxel, gemcitabine and doxorubicin. Commonly-used materials in the nanoparticle-engineered MSCs include polymeric micelles, mesoporous silica, dendrimers and PLGA (Li et al., 2011 ▶; Tripodo et al., 2015 ▶; Wang et al., 2018 ▶). Incorporation of GBA into PLGA NPs increases drug-loading capacity of MSCs warranting that a therapeutic dose of the GBA is released at the tumor site (Vallet-Regí et al., 2018 ▶). Wang et al. loaded bone-marrow-derived MSCs with paclitaxel (PTX) -PLGA NPs and explored their application against glioma cancer in the Transwell system (*in vitro*) and rats (*in vivo*). The PTX-PLGA NP-loaded MSCs treatment increased sustained PTX release in both form of free paclitaxel and paclitaxel NPs. In addition, as expected, the survival time of orthotropic brain-tumor rats compared to free PTX or PXT- PLGA increased (Wang et al., 2018 ▶). In our investigation, PLGA NPs containing GBA were prepared by single emulsion solvent evaporation method. The loading efficiency (EE %) and drug loading (LE%) of GBA in PLGA NPs were 71 and 3.9%, respectively. The loading efficiency of GBA in PLA-PEG NPs was about 40% of that reported by Afsharzadeh et al. (Afsharzadeh et al., 2019 ▶). 

PLGA NPs displayed an initial release about 18% in the first 4 hr at pH 7.4, while it was about 40% at pH 5.5. After 24 hr, around 80% of GBA was released from PLGA NPs at pH 5.5, while it was about 21% at pH 7.4 followed by steady release until 120 hr when the GBA release was about 50%, suggesting that it has good stability in blood circulation. The initial burst has been reported in other studies to be due to the hydrophobic drug molecules located on or near the surface of the NPs.

Drug released from PLGA/GBA NPs loaded hMSCs (nano-engineered hMSCs) during 72 hr was 45.06 and 29.80% for 20 and 40 µM, GBA-equivalent, respectively followed by sustained release of GBA approximately 60 and 50% in 144 hr. These results could ensure the sustained release of GBA from nano-engineered hMSCs into systemic circulation. 

It is important that loading of GBA-containing NPs does not reduce hMSCs viability or their properties including migration ability. The cytotoxicity of GBA and PLGA/GBA against hMSCs was also evaluated by MTT test. Results showed that PLGA/GBA NPs were non-toxic to hMSCs at different concentrations of 1.25-40 µM (GBA-equivalent) as the majority of hMSCs were viable subsequent to loading. Viability of hMSCs was increased when treated with PLGA-encapsulated GBA compared to free GBA. This is in good agreement with other reports that MSCs loaded with NPs maintained their viability and inherited characteristic such as proliferation, migration and tumor-localizing capacity (Paris et al., 2016 ▶; Paris et al., 2017 ▶; Labusca et al., 2018 ▶). In next step, the migration capacity of nano-engineered hMSCs, as an important MSCs property, toward cancerous C26 cells and normal NIH/3T3 cells was evaluated. Surprisingly nano-engineered hMSCs had higher ability to infiltrate C26 cells in comparison to naive hMSCs. Our results shared a number of similarities with Wang et al study (Wang et al., 2018 ▶). Their study revealed that there was no significant difference between the numbers of migratory MSCs treated with low-concentration PTX-PLGA NPs and unloaded MSCs.

The ability of nano-engineered hMSCs to suppress cancer cells was evaluated in colon carcinoma cell line (C26 cells). Nano-engineered hMSCs could effectively induce cell death in C26 cells compared to non-engineered hMSCs, indicating that MSCs as a cellular vehicle had no effect on inhibiting or promoting cancer cells growth. It can be expected that this cellular carrier could efficiently target tumor cells in animal models of cancers and increase tumor homing. This is consistent with previous studies demonstrating that nano-engineered hMSCs resulted in greater tumor inhibition in different types of cancers (Yao et al., 2017 ▶; Zhao et al., 2017 ▶; Wang et al., 2019 ▶). 

Although long-term studies are needed for further evaluation of this system's efficacy, our current study showed that nano-engineered hMSCs had great ability to migrate toward cancer cells and they can serve as an efficient cellular carrier for targeted drug delivery to tumor cells. Future* in vivo* studies can investigate the efficacy of these carriers in animal tumor models. 

In the present study, MSCs were isolated from human adipose tissue and for the first time, and loaded with GBA containing PLGA NPs (PLGA/GBA NPs) to construct a cellular carrier to suppress cancer cells. The viability of hMSCs and their important feature, ability to migrate toward cancer cells, were found unaffected after PLGA/GBA loading. hMSCs carrying PLGA/GBA NPs (nano-engineered hMSCs) were shown to be efficient in killing C26 colon cancer cells *in vitro* in a dose-dependent manner. 

Our study indicated that nano-engineered hMSCs could be considered a promising cellular carrier for targeted delivery of anti-cancer therapeutics.

## Conflicts of interest

The authors have declared that there is no conflict of interest.
